# DNA methylation of insulin-like growth factor 2 and *H19* cluster in cord blood and prenatal air pollution exposure to fine particulate matter

**DOI:** 10.1186/s12940-020-00677-9

**Published:** 2020-12-07

**Authors:** Congrong Wang, Michelle Plusquin, Akram Ghantous, Zdenko Herceg, Rossella Alfano, Bianca Cox, Tim S. Nawrot

**Affiliations:** 1 Centre for Environmental Sciences, Hasselt University, Agoralaan gebouw D, 3590 Diepenbeek, Hasselt, Belgium; 2 Epigenetics Group, International Agency for Research on Cancer (IARC), Lyon, France; 3 Department of Public Health and Primary Care, Leuven University, Leuven, Belgium

**Keywords:** Imprinted genes, *IGF2*, *H19*, Methylation, Factor analysis, DLNM, Air pollution, PM_2.5_, Gestation, Newborn, Fetal growth

## Abstract

**Background:**

The *IGF2* (insulin-like growth factor 2) and *H19* gene cluster plays an important role during pregnancy as it promotes both foetal and placental growth. We investigated the association between cord blood DNA methylation status of the *IGF2/H19* gene cluster and maternal fine particulate matter exposure during fetal life. To the best of our knowledge, this is the first study investigating the association between prenatal PM_2.5_ exposure and newborn DNA methylation of the *IGF2/H19*.

**Methods:**

Cord blood DNA methylation status of *IGF2/H19* cluster was measured in 189 mother-newborn pairs from the ENVIR*ON*AGE birth cohort (Flanders, Belgium). We assessed the sex-specific association between residential PM_2.5_ exposure during pregnancy and the methylation level of CpG loci mapping to the *IGF2*/*H19* cluster, and identified prenatal vulnerability by investigating susceptible time windows of exposure. We also addressed the biological functionality of DNA methylation level in the gene cluster.

**Results:**

Prenatal PM_2.5_ exposure was found to have genetic region-specific significant association with *IGF2* and *H19* during specific gestational weeks. The association was found to be sex-specific in both gene regions. Functionality of the DNA methylation was annotated by the association to fetal growth and cellular pathways.

**Conclusions:**

The results of our study provided evidence that prenatal PM_2.5_ exposure is associated with DNA methylation in newborns’ *IGF2/H19*. The consequences within the context of fetal development of future phenotyping should be addressed.

**Supplementary Information:**

The online version contains supplementary material available at 10.1186/s12940-020-00677-9.

## Background

Genomic imprinting is an epigenetic process leading to monoallelic gene expression and one of the major regulators of genomic imprinting is DNA methylation. Imprinted genes are involved in cellular pathways crucial for growth and development [1]. Among over a hundred imprinted genes in humans, a pair of widely investigated is the insulin-like growth factor 2 (*IGF2*) gene, clustered with the reciprocally imprinted neighboring *H19* gene. With one of the two alleles silenced, the paternal allele of *IGF2* gene is expressed and the maternally active *H19* gene downstream to *IGF2* is transcribed into a non-coding RNA. Studies have shown *IGF2* as a contributor to maternal nutrient supply to the fetus [2] and that loss of imprinting in *IGF2* resulted in fetal overgrowth [3]. *H19* partly serves as a regulator of *IGF2* expression [4]. The DNA methylation levels in differentially methylated regions (DMR) of these two imprinted genes in placenta and cord blood have been reported to associate with birth size [5–8].

Ambient airborne fine particulate matter with diameter smaller than 2.5 μm, PM_2.5_, is one of the air pollution components with the strongest adverse effects on health and mortality as it can penetrate the respiratory system, circulate via bloodstream to other organs [9, 10] and cross the maternal–fetal placental barrier [11, 12]. Epidemiological studies have shown that maternal exposure to PM_2.5_ is associated with preterm birth and low birth weight [13, 14], elevated blood pressure [15, 16], changes in heart rate variability [17], respiratory disease [18] and central nervous system diseases [19, 20]. The imprinting status of genes is susceptible to environmental changes especially during fetal development, when DNA synthesis and cell division are extremely active. Prenatal exposure to PM_2.5_ may have even life-long consequences as, according to the Developmental Origins of Health and Diseases (DOHaD) theory, perturbations in the intrauterine environment are involved in the development of disease in later life [21]. Alterations in methylation of the imprinted *IGF2/H19* cluster might be a potential mechanism underlying the association between in utero PM_2.5_ exposure and fetal growth, as maternal residential PM_2.5_ has been reported to alter their expression [22]. In this study, we assessed the association between maternal PM_2.5_ exposure during pregnancy and the DNA methylation level specific to *IGF2/H19* gene cluster in cord blood collected at birth.

## Methods

### Study population

From the ongoing population-based prospective ENVIRONmental influence *ON* AGEing in early life (ENVIR*ON*AGE) birth cohort [23], 199 mother-newborn (all singleton) pairs that were recruited between July 2014 and June 2015 were included. The study has been approved by the ethical committees of Hasselt University and East-Limburg Hospital and was conducted according to the ethical principles in Helsinki declaration [24]. Owing to missing exposure measurements (*n* = 2) and unavailable covariate information (*n* = 8), the final sample size in model fitting was 189.

The ENVIR*ON*AGE birth cohort recruits mothers with a singleton newborn at arrival for delivery at the East-Limburg Hospital. At the first antennal visit (weeks 7–9 of pregnancy), maternal body mass index (BMI) was determined and the date of conception was estimated on the basis of the first day of the mother’s last menstrual period combined with the first ultrasonographic examination. We collected detailed information as newborn’s sex, gestational age, birth date, maternal age and parity from medical records. Ethnicity of a newborn was classified as European if at least 2 grandparents were Europeans and classified as non-European otherwise. Educational level of the mothers was coded “low” if they did not obtain a high school diploma, as “middle” if they obtained a high school diploma, and as “high” if they obtained a college or university degree. Maternal smoking status was categorized as “never smoked”, “former smoker” (when having quit smoking before pregnancy), or “smoker” (in case of continuing smoking during pregnancy). The presence of pregnancy complications was obtained from medical records on gestational diabetes, hypertension, hyperthyroidism or hypothyroidism, infectious disease, preclampsia, vaginal bleeding, phenylketonuria and allergy or asthma. Birth weight and length of newborns were also collected, based on which the Ponderal index (PI), an indicator of fetal growth status, was calculated according to Rohrer’s formulas: PI = 100 × Birth weight (in grams) / [Birth length (in centimeter)]^3^ [25].

### PM_2.5_ exposure assessment

Daily PM_2.5_ concentration (μg/m^3^) measurements were obtained from the Belgian Interregional Environment Agency. Residential PM_2.5_ concentrations of the mothers during pregnancy was estimated for each mother’s address by the combination of land-cover data from satellite images and pollutant data from official fixed monitoring stations, processed by a model chain of spatial-temporal interpolation [23] and a dispersion model [26], providing high-resolution daily exposure values (on 100 m grids). Address changes during pregnancy were considered. In the Flemish region, this interpolation model predicts 80% of the spatial and temporal variance (based on R^2^) [27], and was validated with measurements of internal exposure in urine [10] and for gestational exposure with placental carbon load [11]. Based on the daily residential PM_2.5_ concentrations, we calculated the weekly mean PM_2.5_ concentrations of gestational week 1 to week 40 for each mother, with week 1 starting from the estimated date of conception. In case that gestational age was less than 40 weeks, the exposure values after delivery until week 40 were set to zero.

### DNA methylation measurement

Details on cord blood sample collection are provided in Method S1, Additional file 1. DNA methylation was measured at the International Agency for Research on Cancer of Lyon in France. DNA extracted from buffy coat of cord blood samples collected at birth were used to determine the epigenome-wide DNA methylation profile through hybridization to the Illumina Infinium HumanMethylation450K BeadChip arrays [28]. The methylated (M) and unmethylated (U) signal intensities were detected and processed in R using the *minfi* package [29] to calculate the beta-value indicating the methylation level at each CpG site: β = M/(M + U + α), with α = 100 an offset value used to stabilize the calculation when both M and U are small [30]. The resulting beta-values, ranging from 0 (unmethylated) to 1 (fully methylated), were exported for quality control and pre-processing. The methylation data was filtered from cross-reactive probes and low-quality probes (probes with bead counts lower than 3 in at least 5% samples). Data quality was further evaluated by checking the distribution of the methylated and unmethylated signals. Sample outliers and gender mismatches were removed based on multidimensional scaling plots and the results of unsupervised clustering. Samples which had more than 1% of the probes with detection *p*-value > 0.05 were removed. The remaining DNA methylation data (485,577 probes) underwent functional normalization [31] using the *minfi* package. Each CpG locus was trimmed for potential outliers based on the range [Q_1_–3 × IQR, Q_3_ + 3 × IQR], with Q1 and Q3 the first and the third quartiles respectively, and IQR the inter-quartile range. Beta-values identified as outliers were replaced by missing values. Additionally, 40,590 probes targeting non-specific CpGs, 15,702 probes with missing values in over 20% of the samples, and 11,648 probes located on X or Y chromosomes were excluded. The remaining probes were examined if they contained single nucleotide polymorphisms located within 10 bps (SNP < 10 bps) of the target CpG, as suggested by Illumina [32], and the probe filtering for SNP < 10 bps was restricted to those found in newborns [33]. The CpG loci with their UCSC reference gene name referring to *IGF2* or *H19* were selected for the present study. In total, 145 CpGs mapping to *IGF2* gene or its adjacent region including *IGF2*-*INS* and 62 CpG loci mapping to *H19* were selected. Imputation methods were not applied because they tend to modify the correlation structure [34] and the amount of missing values was not substantial (Method S2, Additional file 1). We therefore excluded the CpG loci with incomplete records. 109 CpG loci of *IGF2* and 53 of *H19* remained for further analysis.

### Statistical modelling

The beta-values and correlation structure of the CpGs mapping to each one gene was visualized using R package ComplexHeatmap [35]. In order to address the inter-correlation of the CpG loci [36], reduce the number of independent hypothesis tests and remove less relevant CpG loci, we performed factor analysis on the CpG loci mapping to *IGF2* and *H19*, respectively. Factor analysis was performed based on the correlation matrices using iterative principal component factoring and varimax rotation, maximizing the sum of the squared loadings. Parallel analysis [37, 38] was used to decide the number of common factors to extract. The extracted factors were orthogonal to each other and the standardized factor scores were used as integrated measures for the actual methylation beta-values of the CpG loci and each entered a regression model as the response variable. CpG loci were selected as relevant to a factor if the absolute value of their factor loadings were larger than 0.45, which was visualized in chord diagrams using R package circlize [39]. Since the methylation array was performed in batches with 2 × 6 samples per each array chip, batch effects existed due to sample plate and sentrix positions (row- and column-coordinates). At least one of these grouping categories were introduced into the analysis as random effects, based on test results from the exact likelihood ratio test (LRT) for the presence of random effects for each of the *IGF2* and *H19* factors (Method S3, Additional file 1).

The models were adjusted for covariates chosen a priori based on previous studies [40, 41]. Those were parameters characterizing the mother and newborns: maternal age, pre-pregnancy BMI, educational level, smoking status, parity and presence of any pregnancy complication; newborn’s sex, gestational age, birth date, birth season and ethnicity. All categorical variables were contrast-coded, and all continuous variables except birth date were centered around the mean. The date of birth was calculated as the time difference in days between the actual birth date of each newborn and the first birth date in this data set. An interaction between newborn’s sex and PM_2.5_ exposure was included in the models based on previous evidence of sex-specific differences on molecular level in response to prenatal environmental exposures [42, 43]. Whether newborn’s sex was an effect modifier was assessed by performing a likelihood ratio test (LRT) on the interaction term. Afterwards, for each sex-group the change of factor scores for a 5 μg/m^3^ increment in PM_2.5_ concentration was estimated at each gestational week using distributed lag nonlinear models (DLNMs) proposed by Gasparrini et al. [44]. The DLNMs provide a flexible method to model the level of exposures while adjusting for lagged exposure values and thereby allows the identification of vulnerable exposure windows, which in turn provides hints on mechanisms through which exposure acts on fetal health [41, 45]. The exposure-response relationship and lag-response relationship are simultaneously involved in one model, via the construction of a cross-basis combining two basis-functions corresponding to exposure structure and lag structure, respectively. We assumed the exposure-response relationship to be linear and specified for the lag structure a natural cubic spline with 3 inner knots equally spaced along the original lag scale (week 1 to week 40) based on a previous study [41]. The total degree of freedom (DF) of the cross-basis was 5. The association between the factors and prenatal PM_2.5_ exposure was estimated for each gestational week. Based on the same DLNM models, the cumulative association over the entire pregnancy as well as for each trimester was calculated as the incremental cumulative predicted associations from gestational week 1 to week 40, from gestational week 1 to week 13, from gestational week 14 to 26, and from gestational week 27 to 40, respectively.

Since the maximum likelihood (ML) or restricted maximum likelihood (REML) estimators used in mixed model estimation are not robust to outliers, we applied a robust estimator using a smoothed Huber ψ-function and squared robustness weights. This method allows controlling the robustness on single observation level as well as on the group level. For the main analysis we had the estimator’s efficiency fixed at 95% relative to REML. This was done by setting the tuning parameter k for both fixed and random effects at 1.345 [46]. When likelihood needed to be calculated for model selection or LRT, the ML- or REML-estimator was used instead of the robust estimator.

In sensitivity analyses, (1) we excluded all pre-term birth observations to lower the potential influence of the missing PM_2.5_ measurements entering the model as zeros; (2) the flexibility of the natural cubic spline function for modelling lag structure was varied so that the total DF of cross-basis was compared between DF = 5 and DF = 7 or DF = 9; in parallel, an unconstrained DLM was fitted where all 40 weekly mean exposures entered the model separately; (3) the choice of robustness and estimation efficiency of the estimator was assessed, by comparing the main results (k = 1.345) to a non-robust REML estimator (k = ∞) and to a more robust but less efficient estimator (k = 1.69).

In order to address the functionality of DNA methylation in these two genes, we correlated the factor scores to cord blood DNA transcriptome. The detailed procedure of profiling the transcriptome, quality control, normalization and preprocessing is provided in Method S4, Additional file 1. In total, pairwise Pearson correlation was calculated between each factor and 29,164 transcripts. Based on the ascendingly ordered p-values of the correlation tests which were smaller than 0.05, the first 100 transcripts were used to perform the overrepresentation analysis (ORA) using the R package ReactomePA [47]. A pathway was considered significantly enriched if the p-value was smaller than 0.01 and q-value was smaller than 0.05 with at least 3 genes included in the functional set of a pathway. In addition, we assessed the association between the methylation level and fetal growth by regressing newborn’s birth weight or PI on the factor scores. The birth weight as well as PI were surrogates of fetal growth which have been reported to associate with changes in expression level of growth- and development-related genes [48]. The regression models were adjusted for maternal age, pre-pregnancy BMI, educational level, smoking status, parity, presence of pregnancy complications, newborn’s sex, gestational age, birth date, birth season and ethnicity. We assumed causality for the association among PM_2.5_ exposure, DNA methylation and fetal growth, and performed mediation analysis [49] to assess whether the change in *IGF2/H19* DNA methylation mediates the association between prenatal PM_2.5_ exposure and fetal growth.

Data analyses were conducted in R (version 3.6.0) and SAS 9.4 (SAS Institute, Cary NC). Based on the number of extracted factors per gene, the family wise error rate (FWER) was controlled below 0.05 using the Sidak correction.

## Results

### Descriptive statistics

**Fig. 1:**
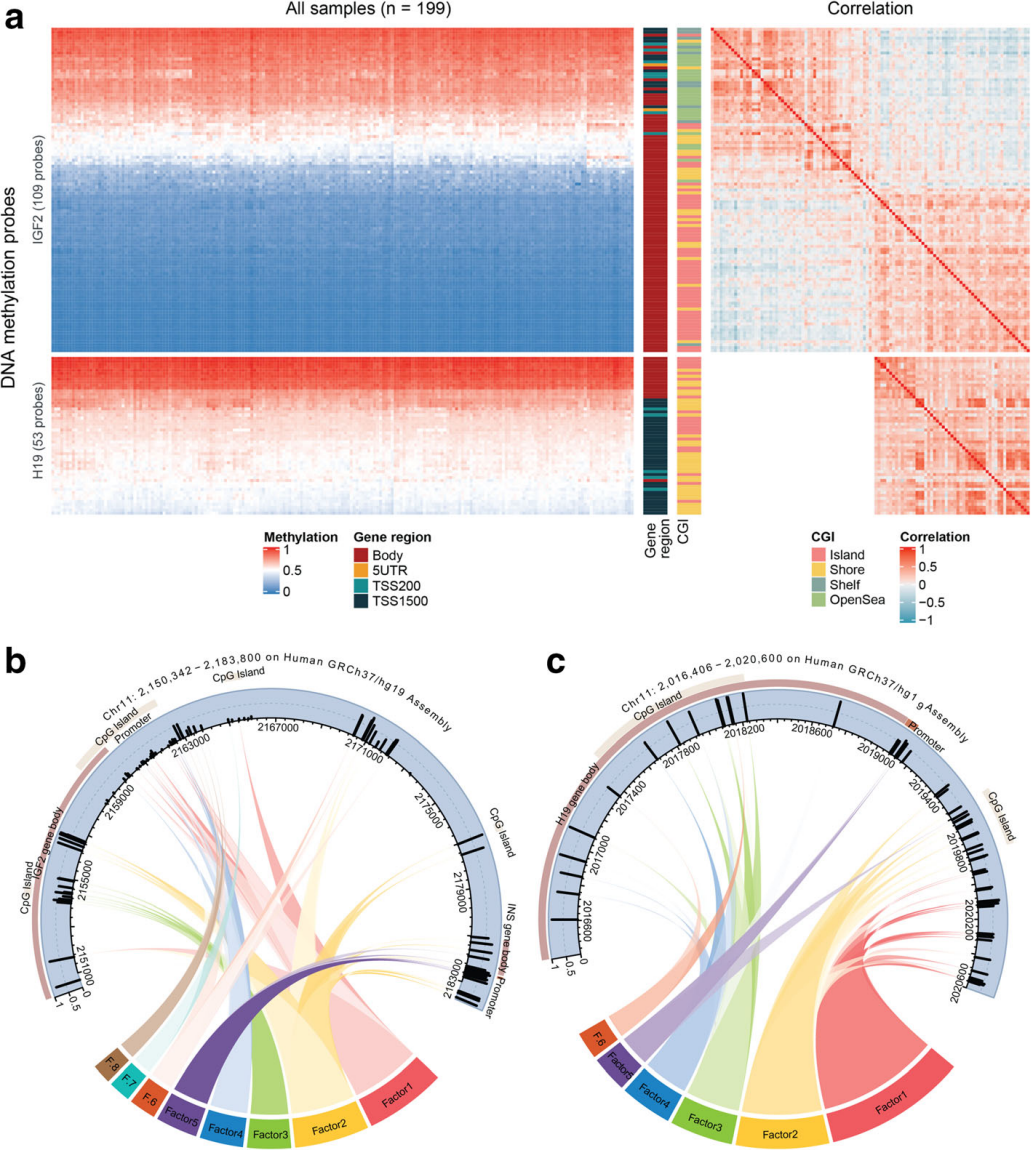
Heatmaps of the CpGs and cord diagrams of the CpGs and factors. **(a).** Heatmaps of the 109 *IGF2*-related CpGs (upper rows) and 53 *H19*-related CpGs (lower rows). From left to right panels: heatmap of the beta-values, gene region annotation of the CpGs, CpG island annotation of the CpGs and correlation heatmap of the pairwise Pearson correlations. **b**. *IGF2* CpGs and **c** *H19* CpGs shown with beta-value averaged over all observations and their genomic context (obtained via UCSC Genome Browser). Factors were shown in different colors and the width represents the relative amount of variance explained by each factor. "F." is the abbreviation of "Factor". Factors and CpGs were connected with colored ribbons representing the factor loadings, where only loadings with absolute value larger than 0.45 were shown and higher color saturation indicates higher value of the loading. Except the loading of cg26913576 (the second CpG from left in **(B)**) on *IGF2* Factor 1 was − 0.62, all loadings shown in these two diagrams were positive

Details of the characteristics of the mother-newborn pairs are summarized in Table 1. 91 of the 189 newborns were girls (48.1%). PI of all newborns was 2.69 ± 0.22 g/cm^3^. 14 newborns (7.4%) was born preterm (10 boys and 4 girls). The weekly average residential PM_2.5_ concentration of 40 weeks for all mothers is summarized in Table 2, with a global mean of 12.97 and high variability (SD: 8.25 μg/m^3^). The correlation structure of the weekly average PM_2.5_ is shown in a heatmap of pairwise Pearson correlations in Figure S1, Additional file 1. PM_2.5_ levels of adjacent weeks were in general positively correlated. Some of the correlations were relatively higher than others, such as those near the end of gestation. Correlation between non-neighboring gestational weeks mainly appeared in the middle part of gestation. The beta-values as well as the correlation structures of the CpG loci mapping to *IGF2* and *H19* are illustrated in Figure 1a. Using a cutoff value of 0.50 for the beta-value, most of the CpGs resided in *IGF2* gene body and close to CpG island showed hypomethylation, while those neighboring the transcription starting site (TSS) and distant to CpG island were hypermethylated. All 53 *H19* CpGs were inside or close to CpG island and the majority of these CpGs displayed hypermethylation, especially those within gene body. The correlation heatmaps suggested that the hypermethylated loci tended to be highly positively correlated in both sets of CpGs and intercorrelation was also present within the hypomethylated *IGF2* CpGs. This intercorrelation was consistent with the Kaiser-Meyer-Olkin (KMO) measure of sampling adequacy, which was 0.79 for CpG loci of *IGF2* and 0.92 for CpG loci of *H19*, indicating factorability of each of the two sets of CpGs (KMO > 0.50 is considered suitable [50]). Both the heatmaps and KMO also suggested that correlations among all the *H19* CpGs are higher than that among all the *IGF2* CpGs.

**Table 1:** Characteristics of the study population (189 mother-newborn pairs)

Characteristic	N (%)	Mean ± SD
***Newborns***		
*Sex*		
Male	98 (51.9)	
Female	91 (48.1)	
*Ethnicity*		
European	170 (89.9)	
Non-European	19 (10.1)	
*Season of birth*		
Winter	42 (22.2)	
Spring	82 (43.4)	
Summer	26 (13.8)	
Autumn	39 (20.6)	
*Birth weight (kg)*		3.40 ± 0.48
*Ponderal index (g/cm*^*3*^*)*		2.69 ± 0.22
*Gestational age (weeks)*		39.1 ± 1.6
< 37	14 (7.4)	
37	6 (3.2)	
38	26 (13.8)	
39	46 (24.3)	
40	72 (38.1)	
41	25 (13.2)	
*Date of birth (days)*		207 ± 102
***Mothers***		
*Educational level*		
Low	27 (14.3)	
Middle	65 (34.4)	
High	97 (51.3)	
*Smoking status*		
Never smoker	122 (64.6)	
Former smoker	41 (21.7)	
Smoker	26 (13.8)	
*Parity*		
Primiparous	104 (55.0)	
Secundiparous	59 (31.2)	
Multiparous	26 (13.8)	
*With pregnancy complications*	40 (21.2)	
*Age (years)*		29.3 ± 4.3
*Pre-pregnancy BMI (kg/m*^*2*^*)*		24.2 ± 4.2

**Table 2:** Summary statistics of weekly average PM_2.5_ concentration in μg/m^3^

Mean ± SD	Minimum	1st quartile	Median	3rd quartile	Maximum
12.97 ± 8.25	1.89	7.29	10.74	16.34	91.30

The missing weekly average PM_2.5_ values that were imputed as zero were excluded

### Constructing integrated cord blood methylation measures of *IGF2* and *H19*

An 8-factor solution and a 6-factor solution were used to construct the factor models for *IGF2* and *H19,* respectively. When assessing the factor solution, we referred to the amount of total variance explained by the factors (higher than 60% as acceptable) and the square root of mean squared off-diagonal residuals (RMSR) (smaller than 0.042 as acceptable according to Kelly’s criterion). The 8 factors of *IGF2* accounted for 51.2% of the total variance of the 109 CpGs and the corresponding RMSR was 0.04. Within the explained variance, the proportions of variance explained by Factor1–8 were 23.0, 21.1, 12.4, 12.3, 11.8, 7.7, 6.2 and 5.5%, respectively. For *H19* gene, the 6 factors explained 64.0% of the total variance of the 53 CpGs and the model RMSR was 0.03. Factor1–6 covered 34.3, 22.5, 15.2, 12.6, 8.7 and 6.6% of the explained variance, respectively. After varimax rotation of the factor axes, the standardized factor scores were obtained, which ranged from − 3.79 to 4.54 in the 8 *IGF2* factors and from − 3.76 to 3.02 in the 6 *H19* factors. Factor loadings were shown in Table S1 and S2, Additional file 2. For each factor, the CpG loci with the absolute value of factor loading higher than 0.45 were grouped to that factor and are shown in the chord diagrams in Fig. 1b and c. All these factor loadings shown in the chord diagrams had a positive value, except that the loading of cg26913576 on *IGF2* Factor1 was − 0.62. *IGF2* Factor1, Factor4, Factor7 and Factor8 mostly pointed to a hypomethylated region near the *IGF2* promoter and Factor 2 covered several genomic regions which were hypermethylated. Factor3 was mostly linked to CpGs inside *IGF2* gene body and Factor5 was mostly related to the *INS* gene. *H19* Factor1, Factor2 and Factor 5 mainly related to the CpGs outside *H19* gene body and Factor5 also pointed to the promoter region. In the meanwhile, *H19* Factor3, Factor4 and Factor6 were linked with CpGs within *H19* gene body, which showed a relatively higher methylation level.

The standardized factor scores of the factors were used as response variables in the models, hence 8 independent hypotheses were tested for *IGF2* and 6 independent hypotheses were tested for *H19*. By controlling the FWER at 0.05 for each gene using Sidak correction, the individual confidence level for *IGF2* was 99.36% and for *H19* was 99.15%. In sex-stratified analysis, the corresponding confidence levels were 99.68 and 99.57% due to the doubling of number of hypothesis tests.

### Integrated cord blood DNA methylation levels of *IGF2* and *H19* in association with prenatal PM_2.5_ exposure

**Fig. 2:**
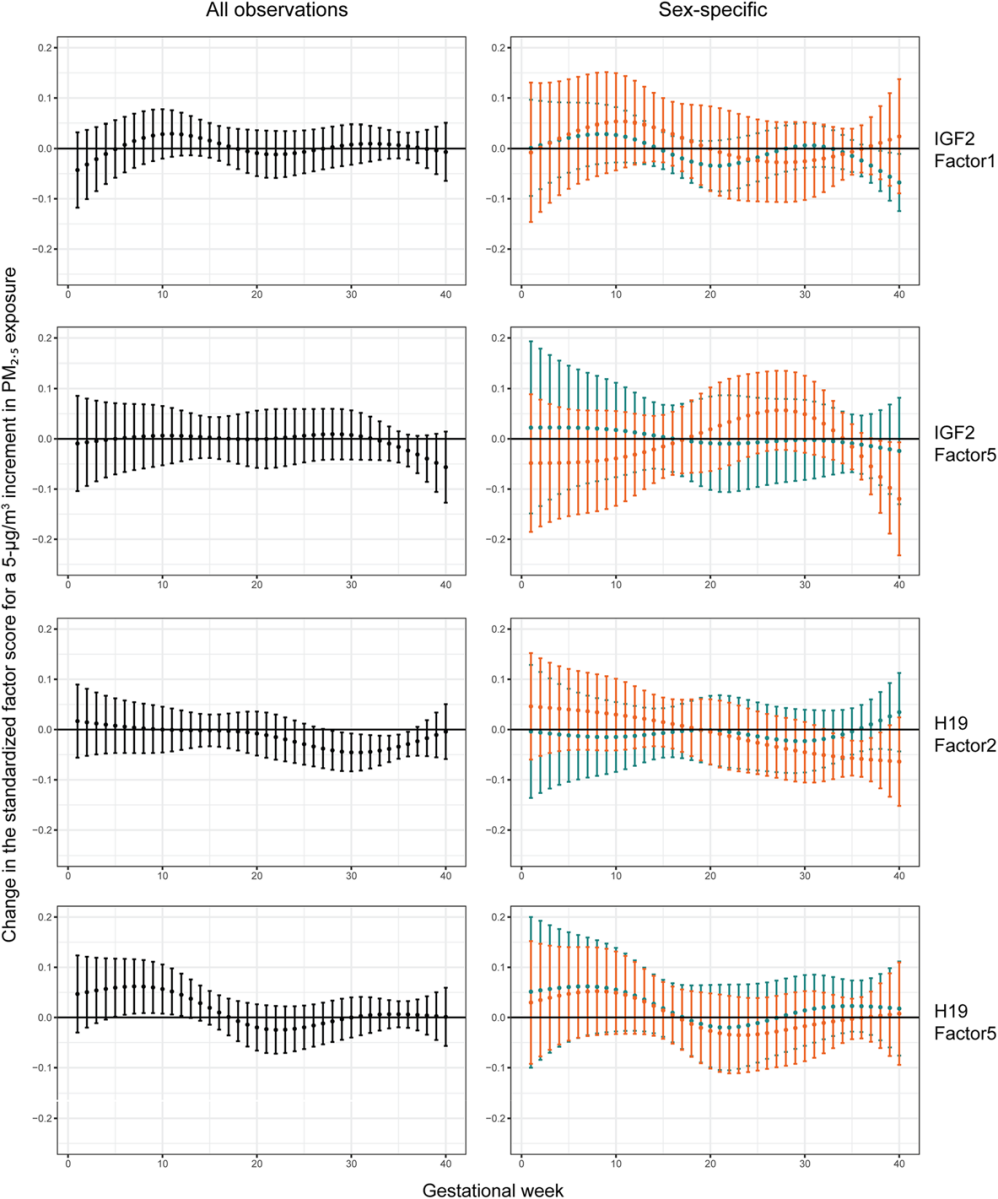
DLNM estimates of the week-specific associations. The estimates were in all observation (left column, *n* = 189), specific to newborn boys (*n* = 98, green) or girls (*n* = 91, orange) (right column), for *IGF2* Factor1 (row 1) and Factor5 (row 2), and *H19* Factor2 (row 3) and Factor5 (row 4), respectively. Estimates are presented as the change in factor scores for a 5-μg/m^3^ increase in PM_2.5_ concentration. Whiskers around the point estimates represent the confidence intervals of the week-specific estimates. For *IGF2* factors, confidence level is 99.36% for all-observation and 99.68% for sex-specific analysis. For *H19* factors, confidence level is 99.15% for all-observation and 99.57% for sex-specific analysis

The interaction between newborn’s sex and PM_2.5_ cross-basis was significant in *H19* Factor2 (LRT *p*-value = 0.036) indicating the effect of PM_2.5_ on methylation being sex-specific. Although the effect modification was not detected in other *H19*-factors and *IGF2*-factors (Table S3 and S4, Additional file 1), we report our results for all observations as well as in a sex-stratified way. Only the results for *IGF2* Factor1 and Factor5 and *H19* Factor2 and Factor5 were displayed because in other factors no significant associations were found. Figure 2 shows the week-specific estimates of the association between maternal exposure to PM_2.5_ during pregnancy and the standardized factor scores with confidence intervals. Table 3 shows the cumulative associations over the entire gestation as well as the three trimesters. All of the *IGF2*-factors were not found to be associated with PM_2.5_ exposures when all observations were considered together. However, specific to sexes, *IGF2* Factor1 was significantly inversely associated with PM_2.5_ exposure in only boys during gestational week 38–40 and *IGF2* Factor5 was significantly inversely associated with PM_2.5_ exposure in only girls during gestational week 38–40. For *H19* gene*,* Factor2 was inversely associated with PM_2.5_ exposure during weeks 28–36 in all observations and during week 33–38 only in girls. In addition, the estimated cumulative change per 5-μg/m^3^ increment of PM_2.5_ in *H19* Factor2 over trimester 3 was − 0.46 (99.15% CI: [− 0.79, − 0.13]) in all observations and − 0.73 (99.57% CI: [− 1.20, − 0.26]) in girls. *H19* Factor5 showed a significant positive association with PM_2.5_ exposure during week 5–12 with a trimester-specific cumulative effect over trimester 1: 0.70 (99.15% CI: [0.05, 1.35]) per 5-μg/m^3^ increment of PM_2.5_ in all observations.

**Table 3:** The DLNM estimates of the cumulative association

	Exposure window	All newborns	Boys	Girls
***IGF2*** **Factor1**	Overall	0.11 (− 0.71, 0.94)	− 0.25 (− 1.15, 0.64)	0.35 (− 1.11, 1.81)
	Trimester 1	0.07 (− 0.57, 0.71)	0.25 (− 0.52, 1.01)	0.44 (− 0.79, 1.67)
	Trimester 2	− 0.02 (− 0.49, 0.45)	− 0.27 (− 0.79, 0.24)	0.04 (− 0.81, 0.89)
	Trimester 3	0.06 (− 0.30, 0.41)	− 0.23 (− 0.68, 0.21)	− 0.13 (− 0.73, 0.48)
***IGF2*** **Factor5**	Overall	− 0.14 (− 1.11, 0.83)	0.06 (− 1.52, 1.64)	− 0.40 (− 1.83, 1.02)
	Trimester 1	0.02 (− 0.77, 0.81)	0.25 (− 1.10, 1.60)	− 0.55 (− 1.75, 0.65)
	Trimester 2	0.03 (− 0.55, 0.61)	− 0.06 (− 1.02, 0.90)	0.24 (− 0.60, 1.09)
	Trimester 3	− 0.19 (− 0.61, 0.24)	−0.13 (− 0.93, 0.67)	−0.09 (− 0.69, 0.51)
***H19*** **Factor2**	Overall	−0.54 (− 1.29, 0.20)	−0.29 (− 1.47, 0.89)	−0.34 (− 1.42, 0.74)
	Trimester 1	0.07 (−0.54, 0.68)	− 0.15 (− 1.15, 0.86)	0.46 (− 0.45, 1.37)
	Trimester 2	−0.16 (− 0.60, 0.29)	−0.08 (− 0.80, 0.64)	−0.07 (− 0.73, 0.59)
	Trimester 3	−0.46 (− 0.79, − 0.13)	−0.06 (− 0.71, 0.58)	−0.73 (− 1.20, − 0.26)
***H19*** **Factor5**	Overall	0.61 (−1.19, 1.42)	0.87 (− 0.53, 2.27)	0.23 (− 1.02, 1.48)
	Trimester 1	0.70 (0.05, 1.35)	0.71 (−0.48, 1.90)	0.56 (− 0.49, 1.61)
	Trimester 2	−0.11 (− 0.58, 0.36)	−0.07 (− 0.92, 0.78)	−0.22 (− 0.98, 0.54)
	Trimester 3	0.02 (−0.33, 0.37)	0.23 (− 0.47, 0.94)	−0.11 (− 0.66, 0.43)

The cumulative effect of a 5 μg/m^3^ increase in PM_2.5_ concentration on *IGF2* Factor1 and Factor5 and *H19* Factor2 and Factor5 over different exposure windows. Estimated in all observations (*n* = 189), in boys (*n* = 98) and in girls (*n* = 91), respectively. Confidence intervals are shown with lower and upper bounds. Confidence level for *IGF2* factors in all-observation analysis is 99.36% and that of sex-specific analysis is 99.68%. Confidence level for *H19* factors in all-observation analysis is 99.15% and that of sex-specific analysis is 99.57%

In sensitivity analysis, we repeated the sex-specific associations with exclusion of 14 preterm births (10 boys and 4 girls), which validated our main results since the estimated associations were only slightly more evident if preterm birth observations were excluded (Table S5, Additional file 1). Therefore, there was not much estimation bias induced by setting the missing values to zero at the end of gestation. Sensitivity analyses on the DF of the cross-basis suggested DF = 5 as the best based on model information criterion (Table S6, Additional file 1). In addition, the existence of vulnerable exposure window was confirmed by performing LRT to the unconstrained model where all 40 weekly average exposures entered the model equally. The application of the robust estimator facilitated finding the association while the classical REML estimator failed for *H19* Factor2 (Table S7, Additional file 1).

### Biological indications of the integrated DNA methylation

The pathways that the selected transcripts were mapping to are shown in Figure S2, Additional file 1. The majority of the listed pathways of *IGF2* Factor1 were related hemoglobin metabolism and detoxification of reactive oxygen species. *H19* Factor2 showed relation to the regulation of rRNA expression and the *H19* Factor5 had pathways enriched in cell cycle regulation and response to cellular stress.

We then assessed the association between birth weight or PI, as the surrogate for fetal growth, and each factor that showed significant association with PM_2.5_ exposure (Table S8, Additional file 1). In girls a SD increase in *IGF2* Factor5 is associated with 0.05 g/cm^3^ decrease in PI (*p*-value = 0.026). Mediation analysis was performed assuming the causality in the relationship among PM_2.5_ exposure, *IGF2* Factor5 and PI in girls, despite that PM_2.5_ exposure and PI was not significantly associated, which is tolerable in mediation analysis [49]. We took the average value of the PM_2.5_ concentrations from the last three gestational weeks as the exposure variable since we have found significant association between PM_2.5_ and *IGF2* Factor5 during the last 3 weeks in girls. However, we did not notice the factor scores of *IGF2* Factor5 to be mediating the relationship between fetal growth and air pollution (Table S9, Additional file 1).

## Discussion

Both *IGF2* and *H19* are imprinted genes integrally important in the transfer of nutrients from mother to offspring and involved in fetal and postnatal growth. To assess the importance of the epigenetic regulation of this gene cluster and the influence by in utero particulate air pollution exposure, we tested the association between DNA methylation of the *IGF2/H19* cluster in cord blood and prenatal air pollution and identified vulnerable exposure windows during gestation. The DNA methylation levels in the genes were integrated into common factors and the relationship between the factor scores and exposure was estimated through the DLNM cross-basis. The associations were found to be sex-specific. We also have addressed the functionality and biological significance of the two genes by assessing the association between fetal growth and the methylation levels, and mapping to functional pathways via DNA transcriptome.

Early-life health status have been shown to be associated with methylation and expression of imprinted genes, which are susceptible to environmental changes during fetal growth. Thus, identifying environmental determinants for early-life imprinted gene methylation profile is of importance. A schematic illustration of *IGF2* and *H19* activation is shown in Figure S3, Additional file 1. The two genes share one enhancer downstream of *H19*. DMRs in the promoters of the two genes interact with the enhancer, determining which allele of which gene segment to be transcribed. Studies have shown that methylation on loci in the DMR is important for activating or blocking the transcription [51–54]. No study yet has shown whether the changes in DNA methylation level of the enhancer regions has an influence on molecular pathways or health status in human, although it has been reported that deletion of the enhancer reduced 20% of the birth size in mice [55]. The present study found an association between prenatal PM_2.5_ exposure and methylation of *H19* Factor2 and Factor5, which are factors highly correlated to loci close to the enhancer. In addition, the heatmap in Fig. 1a suggests that *H19* gene body was generally hypermethylated across samples, while the region neighboring the TSS, which included *H19* Factor 2 and Factor5 CpGs, showed much more variation. In the meanwhile, the most variable region in *IGF2* included a fraction of gene body (*IGF2* Factor 5) and a fraction of TSS regions (both *IGF2* factors). Our findings might suggest unrevealed mechanisms underlying the transcription regulation mechanism of *IGF2/H19*.

The CpG loci were found to be highly correlated, calling for multivariate techniques to model the correlation structure. By applying factor analysis to the CpG loci we reduced the dimension of the methylation data and removed CpG loci with low correlations. The extracted factors were independent to each other, allowing the control of FWER. In addition, the varimax rotation of factor axes brought an easier interpretation of the latent factors since each CpG had a high loading on one factor and near zero on other factors, which made the factors to carry non-overlapping information. Factor analysis has been applied to omics data in previous studies [56–58], but the use in an epidemiological study on methylation data is rather new. Most of the CpG loci which mapped to one factor located within each other’s close neighborhood and all factors distributed across the whole length of the genes, justifying our choice of the factors to represent all the CpG loci mapping to the genes.

The direction of the association between PM_2.5_ and *H19* Factor5 was positive while *H19* Factor2, *IGF2* Factor1 and Factor5 were inversely associated with the gestational PM_2.5_ exposure. Since almost all the loadings of the most relevant CpGs on factors were positive, the behavior of factors is consistent in direction with the behavior of the majority of the selected CpG loci, and a positive/negative association of a factor to PM_2.5_ in general could be translated into a local hypermethylation/hypomethylation related to the exposure. This is the situation for *IGF2* Factor5, *H19* Factor 2 and Factor5. An exception was *IGF2* Factor1, which included one CpG (cg26913576) inside gene body with negative loading on this factor. This indicated an inverse correlation between this individual CpG and the other CpGs that were relevant to this same factor, which is also observable in the mean beta-value at cg26913576 (hypermethylated) and the other CpGs (hypomethylated). Therefore, the inverse association between boys’ *IGF2* Factor1 and prenatal PM_2.5_ exposure in weeks 38–40 indicated an inverse association in the majority of CpGs of this factor and simultaneously a positive association at cg26913576. These findings based on factors are only partly comparable to epigenome-wide association studies (EWAS), because although changes in methylation at single CpGs are reflected through the loadings, it is not necessary that individual CpGs are significantly associated with the exposure and the correlation between CpGs is stressed which is not available in EWAS.

Previous studies have shown that placental expression level of *IGF2/H19* was inversely associated with maternal PM_2.5_ exposure during pregnancy [22] and that PM_2.5_ exposure was in association with smaller birth size [14, 40]. It has also been reported that hypermethylation in the DMR of *H19* was associated with lower weight-for-length [59] and higher transcription level of *H19* was linked to larger length-of-gestational-age [60]. Our results of positive correlation between PM_2.5_ exposure and *H19* Factor5, as well as the inverse association between *IGF2* Factor5 and girls’ PI might add to these. On the other hand, the negative association between PM_2.5_ exposure and *H19* Factor2, *IGF2* Factor1 and Factor5 methylation is consistent with a study on smoking showing lower *H19* methylation in cord blood was associated with maternal smoking during pregnancy and partially mediated newborns’ being small for gestational age [61]. In the mediation analysis, we did not identify *IGF2* Factor5 as a mediator significantly in the association between PM_2.5_ exposure and fetal growth in girls. Indeed, the mediation effect should be found in multiple factors since both PM_2.5_ exposure and fetal growth are associated with multi-omic alterations, and *IGF2* Factor5 might take up only a small fraction. However, the regulation pathway of *IGF2/H19* is not completely understood yet. Complexity also comes from that the enhancer is shared by the two genes that are activated in different alleles, but the methylation array provided no clue to distinguish between the methylation on paternal and maternal alleles. Functional interpretation of the factors might find its reflection in the results of pathway analysis. The pathway analysis was performed indirectly via correlating to transcripts in the same cord blood samples, and was expected to suggest potential pathways involved in the epigenetic alteration associated with prenatal PM_2.5_ exposure, which might include *trans* regulation of *IGF2/H19* imprinting, or multi-omic alterations occurred in parallel to DNA methylation. Methylation in the two genes reflected different cellular functions: *IGF2* Factor1 was related to hemoglobin metabolism while *H19* factors were mostly related to cell cycle and response to cellular stress. Through studying the factors of the *IGF2/H19* gene cluster, we might find indication in the influence on metabolism and even cellular ageing [62] by prenatal exposure to fine particulate matters.

The use of DLNM models allowed us to investigate more detailed gestational exposure windows with higher temporal resolution (weeks) while previous studies on exposures during pregnancy mostly focused on trimester averages or on whole pregnancy averages. DLNM has been typically used to investigate the triggering effect of exposures on health outcomes such as mortality, taking delayed effects into account up to, for instance, 1 month after the exposure. Only recently, has the DLNM approach been introduced to prenatal and perinatal epidemiology [41, 63]. The application of robust linear mixed-effects model is also relatively new, through which we reduced the influence of outliers on different levels and improved the detection of the association.

Despite the novelties and strengths of the present study, restrictions existed in the study design since our results are based on pollutant concentration at the maternal residence, and potential misclassification may be present because we could not account for other exposure sources that contribute to personal exposure, such as exposure during commuting or at work. However, our high-resolution model of residential exposure has recently been shown to be associated with internal exposure to nanosized particles black carbon in urine [10] as well as in placental tissue [11]. Moreover, we do not know the persistence of the current findings as our methylation were only studied at one time point (birth). The sample size of this study was relatively small which might have not provided sufficient statistical power. This is especially clear when performing sex-specific analysis. As shown in Fig. 2 for *H19* Factor 5, the associations in all observations, girls and boys were in the same shape. However, the significant association was only detected in all observations but not in either subset.

## Conclusion

Our study showed that alterations in methylation of two imprinted genes known to be important in fetal growth were associated with in utero PM_2.5_ exposure. Our findings added to the growing body of evidence that prenatal exposure to fine particulate matters impacts newborns on molecular level. The identification of vulnerable exposure windows and the specific relevant CpG loci or genetic regions might contribute to further investigation on the mechanism underlying the effects of particulate matters on fetal growth, as well as the unravelling of the molecular pathway of *IGF2/H19* imprinting.

## Supplementary Information

**Additional file 1.** Supplementary Methods S1-S4. Supplementary Tables S3-S9. Supplementary Figures S1-S3.

**Additional file 2.** Supplementary Tables S1-S2.

## Data Availability

Data available on request.

## Abbreviations

BMI: Body mass index
DF: Degree of freedom
DLM: Distributed lag model
DLNM: Distributed lag non-linear model
DMR: Differentially methylated region
DOHaD: Developmental Origins of Health and Diseases
ENVIR*ON*AGE: Environmental Influence on Ageing in Early Life birth cohort
EWAS: Epigenome-wide association study
FWER: Family-wise error rate
IGF2: Insulin-like growth factor 2
LRT: Likelihood ratio test
ML: Maximum-likelihood
ORA: Overrepresentation analysis
PI: Ponderal index
PM: Particulate matter
REML: Restricted maximum likelihood
SD: Standard deviation
TSS: Transcription starting site
