# Supplementary material for: DNA methylation of insulin-like growth factor 2 and H19 cluster in cord blood and prenatal air pollution exposure to fine particulate matter
Source: Environ Health. 2020 Dec 7;19:129. doi: 10.1186/s12940-020-00677-9 (PMC7720562; doi:10.1186/s12940-020-00677-9)
Supplement: Supplementary file 1 — Additional file 1. Supplementary Methods S1-S4. Supplementary Tables S3-S9. Supplementary Figures S1-S3. [file 12940_2020_677_MOESM1_ESM.docx]

**Supplementary methods**

**Method 1: Cord blood tissue collection**

Umbilical cord blood was collected at delivery using BD Vacutainer® plastic whole blood tubes with spray-coated K2EDTA (BD, Franklin Lakes, NJ, USA). Plasma was separated by centrifuging the samples at 3200 rpm for 15 min and the remainder with buffy coat was stored at −80°C until analysis.

**Method 2: Beta-value trimming**

After quality control and preprocessing, outlier trimming was performed for each CpG. The beta-value of a sample was set to missing value (*NA*) if it was outside the range of [Q_1_-3×IQR, Q_3_+3×IQR]. Before trimming, there was no missing value in any of the 145 CpGs mapping to *IGF2* or the 62 CpGs mapping to *H19* gene. After trimming, the number of missing values in these CpGs are summarized as follows:

| Missing conditions | *IGF2* | | | | | | *H19* | | |
| --- | --- | --- | --- | --- | --- | --- | --- | --- | --- |
|  | 0 | 1 | 2 | 3 | 4 | 6 | 0 | 1 | 4 |
| Above Q_3_+3×IQR | 131 | 13 | 1 | 0 | 0 | 0 | 55 | 6 | 1 |
| Below Q_1_-3×IQR | 123 | 13 | 4 | 2 | 1 | 1 | 60 | 2 | 0 |
| Both | 109 | 26 | 5 | 2 | 1 | 1 | 53 | 8 | 1 |

**Method 3: Presence of random effects**

Batch effects in the DNA methylation array included sample plate and sentrix position. The boxplots (not shown) of the 8+6 factors with respect to both grouping variables suggested that outliers existed at both the observation level and the higher group levels. However, when fitting a highly flexible model, such as the robust linear mixed-effects model used in this study, unnecessary random effects might overload the model. Therefore, a preliminary analysis was performed to decide the presence of random effects for each factor using the exact likelihood ratio test (LRT), where the null model was a simple linear model with only fixed effects and the model under the alternative was a simple linear model with either the random sample plate effects or the random sentrix position effects. The exact LRT results suggested model specification with only random sample plate effects for *IGF2* Factor2, 3, 6, 7 and *H19* Factor 1, 2, 4, 5; model with only random sentrix position effects for *IGF2* Factor8 and *H19* Factor3; model with both random effects for *IGF2* Factor 1, 4 and *H19* Factor 6.

**Method 4: Procedure to generate gene expression profiles and data processing**

RNA was extracted using the total RNA miRNeasy mini kit (Qiagen, Venlo, Netherlands) according to the manufacturer’s protocol. Samples were quality checked and further hybridized onto Agilent Whole Human Genome 8×60 K microarrays coupled with Agilent DNA G2505C Microarray Scanner (Agilent Technologies). Scanned images were converted to TXT files using the Agilent Feature Extraction Software (Version 10.7.3.1, Agilent Technologies, Amstelveen, Netherlands). An in-house quality control pipeline developed in R software was used to preprocess raw data by local background correction, omission of controls, flagging of bad spots and spots with too low intensity, log2-transformation and quantile normalization using arrayQC. More information about the flagging and the R-scripts of the pipeline are available at https://github.com/BiGCAT-UM/arrayQC_Module. Further preprocessing included removal of probes showing > 30% flagged data, merging of replicate probes based on the median, and imputation of missing values using K-nearest neighbor imputation (K=15). 29,164 transcripts were left available for further analysis. The transcripts were denoised for batch effect by taking the model residuals regressing the transcripts on hybridization date. Due to the failure in samples for quality control or missing batch information for denoising, 179 samples were left in our analysis, properly aligned with DNA methylation data to match methylation and transcripts within each observation.

**Supplementary Tables**

**Table S3: The p-values of LRT based on classical mixed-effects model testing for effect modifications by sex, for *H19* Factor1 to Factor6.**

|  | **Factor1** | **Factor2** | **Factor3** | **Factor4** | **Factor5** | **Factor6** |
| --- | --- | --- | --- | --- | --- | --- |
| p-value | 0.666 | 0.036 | 0.457 | 0.506 | 0.692 | 0.629 |

**Table S4: The p-values of LRT based on classical mixed-effects model testing for effect modifications by sex, in *IGF2* Factor1 to Factor8.**

|  | **Factor1** | **Factor2** | **Factor3** | **Factor4** | **Factor5** | **Factor6** | **Factor7** | **Factor8** |
| --- | --- | --- | --- | --- | --- | --- | --- | --- |
| p-value | 0.613 | 0.347 | 0.372 | 0.571 | 0.608 | 0.785 | 0.908 | 0.287 |

**Table S5: The cumulative effect of a 5-μg/m^3^ increase in PM_2.5_ concentration on *IGF2* Factor1 and Factor5, *H19* Factor2 and Factor5 over different exposure windows, in all observations (175), boys (n=88) and girls (n=87), respectively. Preterm birth observations (n=14) were excluded. Confidence intervals shown with lower and upper bounds. Confidence level for all-observation analysis is 99.36% in IGF2 factors and 99.15% in H19 factors. Confidence level for sex-specific analysis is 99.68% in IGF2 factors and 99.57% in H19 factors.**

|  | **Exposure window** | **All observations** | **Boys** | **Girls** |
| --- | --- | --- | --- | --- |
| **IGF2**  **Factor1** | Overall | 0.13 [-0.75, 1.02] | -0.36 [-1.31, 0.59] | -0.09 [-1.62, 1.44] |
|  | Trimester 1 | 0.28 [-0.46, 1.02] | 0.18 [-0.70, 1.06] | 0.03 [-1.33, 1.39] |
|  | Trimester 2 | -0.15 [-0.70, 0.40] | -0.32 [-0.91, 0.26] | 0.11 [-0.96, 1.17] |
|  | Trimester 3 | 0.00 [-0.38, 0.38] | -0.22 [-0.69, 0.26] | -0.23 [-0.89, 0.44] |
| **IGF2**  **Factor5** | Overall | -0.14 [-1.17, 0.89] | 0.35 [-1.38, 2.09] | -0.53 [-1.94, 0.88] |
|  | Trimester 1 | 0.11 [-0.78, 1.01] | 0.59 [-1.03, 2.22] | -0.38 [-1.65, 0.88] |
|  | Trimester 2 | 0.01 [-0.43, 0.46] | 0.01 [-1.11, 1.13] | -0.01 [-0.96, 0.94] |
|  | Trimester 3 | -0.25 [-0.70, 0.21] | -0.25 [-1.13, 0.63] | -0.14 [-0.73, 0.46] |
| **H19**  **Factor2** | Overall | -0.43 [-1.21, 0.35] | -0.06 [-1.37, 1.25] | -0.35 [-1.43, 0.73] |
|  | Trimester 1 | 0.22 [-0.45, 0.90] | 0.11 [-1.14, 1.35] | 0.11 [-1.14, 1.35] |
|  | Trimester 2 | -0.14 [-0.65, 0.37] | 0.00 [-0.85, 0.85] | -0.09 [-0.83, 0.64] |
|  | Trimester 3 | -0.51 [-0.85, -0.17] | -0.17 [-0.89, 0.55] | -0.75 [-1.22, -0.29] |
| **H19**  **Factor5** | Overall | 0.58 [-0.28, 1.44] | 1.31 [-0.24, 2.86] | 0.13 [-1.09, 1.36] |
|  | Trimester 1 | 0.77 [0.04, 1.51] | 1.21 [-0.24, 2.66] | 0.71 [-0.38, 1.79] |
|  | Trimester 2 | -0.17 [-0.72, 0.38] | 0.04 [-0.97, 1.04] | -0.42 [-1.27, 0.44] |
|  | Trimester 3 | -0.02 [-0.4, 0.35] | 0.06 [-0.72, 0.85] | -0.15 [-0.69, 0.38] |

**Table S6. Model selection criteria for DLM with different DF in natural cubic spline and unconstrained DLM, based on classical linear mixed model REML estimates, estimated for *H19* Factor2. Analysis performed on all observations.**

|  | **Cross-basis DF** | | | **Unconstrained**  **DLM** |
| --- | --- | --- | --- | --- |
|  | **DF = 5** | **DF = 7** | **DF = 9** |  |
| **Model DF** | 23 | 25 | 27 | 58 |
| **AIC** | 590.03 | 607.45 | 624.60 | 855.52 |
| **LRT p-value comparing**  **to unconstrained DLM** | 0.0011 | 0.0008 | 0.0007 | -- |

**Table S7: The cumulative effect of a 5 µg/m^3^ increase in PM_2.5_ concentration on *H19* Factor2 and Factor5 over different exposure windows in models with different robustness. Analysis performed on all observations. 99.15% confidence intervals shown with lower and upper bounds.**

|  | **Exposure window** | **Classical** | **Robust1** | **Robust2** |
| --- | --- | --- | --- | --- |
| **Factor2** | Overall | -0.26 [-1.04, 0.52] | -0.54 [-1.29, 0.20] | -0.38 [-1.17, 0.40] |
|  | Trimester 1 | -0.02 [-0.63, 0.58] | 0.07 [-0.54, 0.68] | 0.07 [-0.54, 0.68] |
|  | Trimester 2 | 0.02 [-0.42, 0.47] | -0.16 [-0.60, 0.29] | -0.08 [-0.53, 0.36] |
|  | Trimester 3 | -0.26 [-0.61, 0.09] | -0.46 [-0.79, -0.13] | -0.37 [-0.72, -0.02] |
| **Factor5** | Overall | 0.54 [-0.22, 1.30] | 0.61 [-1.19, 1.42] | 0.56 [-0.23, 1.34] |
|  | Trimester 1 | 0.34 [-0.27, 0.94] | 0.70 [0.05, 1.35] | 0.50 [-0.13, 1.13] |
|  | Trimester 2 | 0.01 [-0.43, 0.45] | -0.11 [-0.58, 0.36] | -0.06 [-0.52, 0.39] |
|  | Trimester 3 | 0.19 [-0.15, 0.52] | 0.02 [-0.33, 0.37] | 0.11 [-0.23, 0.46] |

**Table S8. The association between birth weight or PI and the factors that have been found to associate with PM_2.5_ exposure. Estimates are shown with standard errors. The only significant association was found between *IGF2* Factor5 and girls’ PI (p-value = 0.026).**

|  |  | ***IGF2*** | | ***H19*** | |
| --- | --- | --- | --- | --- | --- |
|  |  | Factor1 | Factor5 | Factor2 | Factor5 |
| **All** | Birth weight | -- | -- | -37.3 (29.9) | 29.8 (29.7) |
|  | PI | -- | -- | -0.03 (0.017) | -0.01 (0.017) |
| **Boys** | Birth weight | -0.2 (9.09) | -- | -- | -- |
|  | PI | 0.00005 (0.024) | -- | -- | -- |
| **Girls** | Birth weight | -- | 9.5 (47.0) | 43.5 (50.0) | -- |
|  | PI | -- | -0.05 (0.023) | -0.0007 (0.025) | -- |

**Table S9. The estimated effects from mediation analysis in girls, where *IGF2* Factor5 was the mediator, mean PM_2.5_ concentration of the last 3 weeks was the exposure and girls’ PI was the outcome variable. Estimates shown with 95% confidence intervals.**

| **Effect** | **Estimate** |
| --- | --- |
| **natural direct effect** | 0.026 [-0.027, 0.080] |
| **natural indirect effect** | 0.018 [-0.005, 0.040] |
| **total effect** | 0.044 [-0.006, 0.094] |
| **proportion mediated** | 0.40 |

**Supplementary Figures**


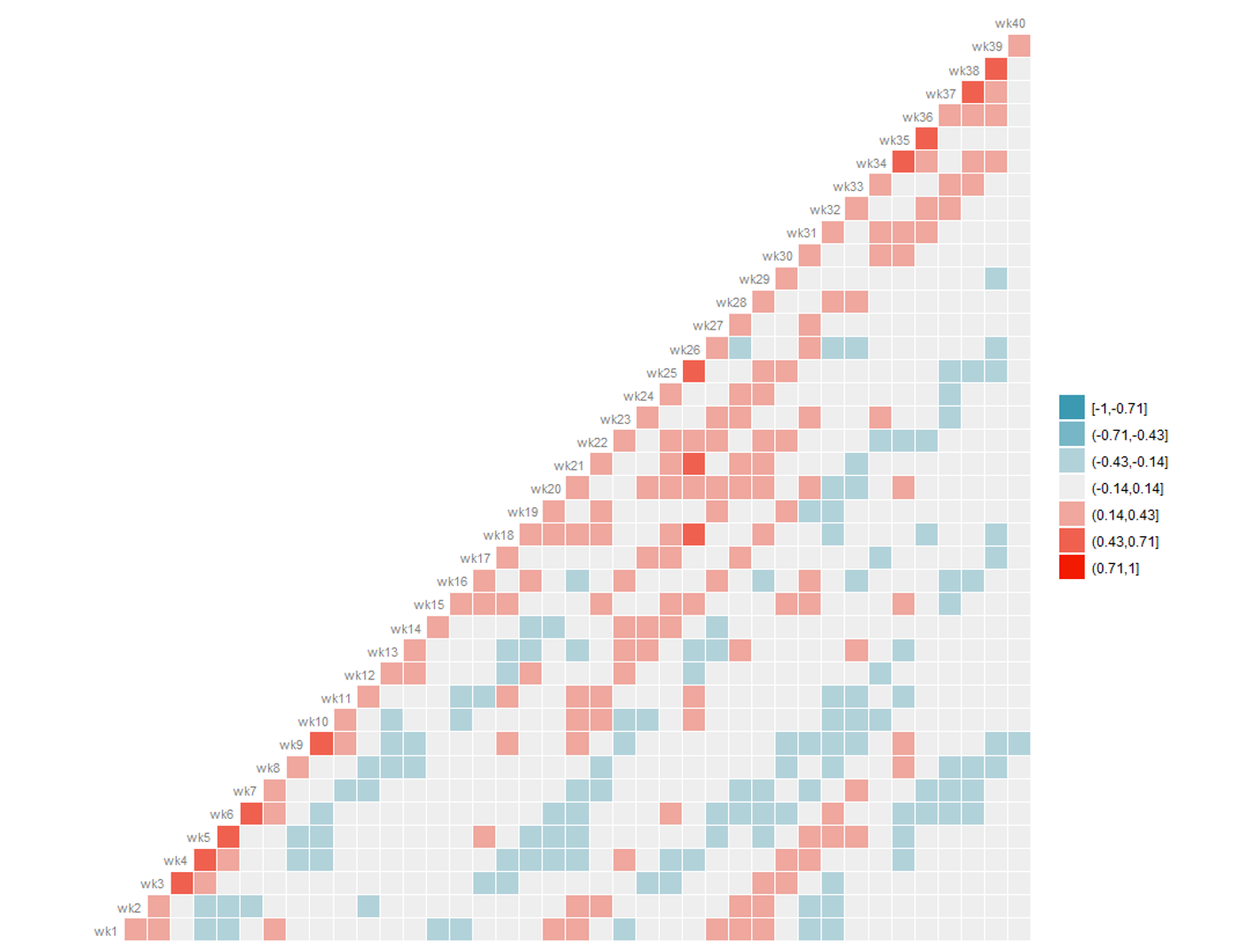


**Figure S1. The pairwise Pearson correlation heat map of weekly average PM_2.5_ concentrations. “Week” was shortened as “wk” in the labels.**


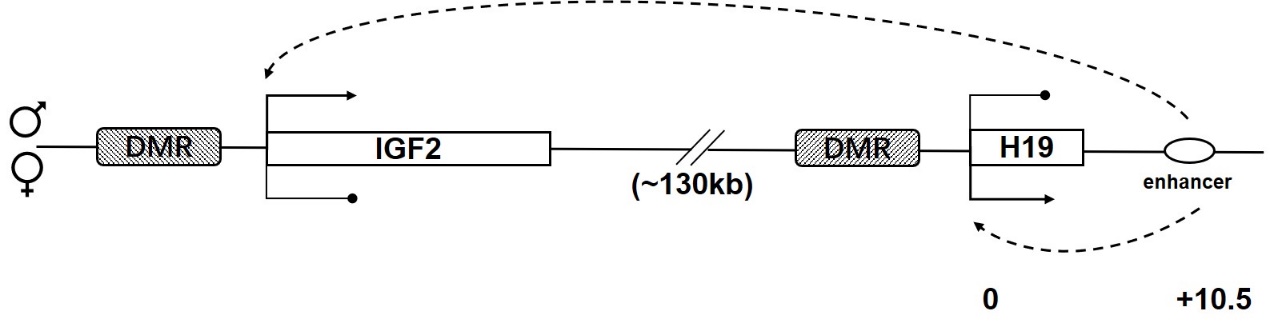


**Figure S4. A simplified model of the *IGF2/H19* gene imprinting. Adapted from [4]. Under normal conditions, the methylation of DMR’s cause the transcription of paternal *IGF2* and maternal *H19*. The numbers below the lines represent the 5′ boundary of regions relative to the *H19* gene transcriptional start site**

**Reference**

1. Franklin, S.B., et al., *Parallel Analysis: A Method for Determining Significant Principal Components.* Journal of Vegetation Science, 1995. **6**(1): p. 99-106.

2. O’connor, B.P.J.B.R.M., Instruments, and Computers, *SPSS and SAS programs for determining the number of components using parallel analysis and Velicer’s MAP test.* 2000. **32**(3): p. 396-402.

3. Valeri, L. and T.J. Vanderweele, *Mediation analysis allowing for exposure-mediator interactions and causal interpretation: theoretical assumptions and implementation with SAS and SPSS macros.* Psychol Methods, 2013. **18**(2): p. 137-50.

4. Pidsley, R., et al., *DNA methylation at the Igf2/H19 imprinting control region is associated with cerebellum mass in outbred mice.* Molecular Brain, 2012. **5**(1): p. 42.
